# Mosquitoes and Yellow Fever

**Published:** 1904-10-15

**Authors:** 


### Mosquitoes and Yellow Fever.

The "Selections from Colonial Medical Reports for 1901 and 1902," just issued from the Colonial Office, contain, among much other matter, some highly interesting "notes" by Mr. St. George Gray, Colonial Surgeon at St. Lucia, on the conveyance of disease by mosquitoes; and, after describing their action in relation to malaria, which is now too well understood to require repetition, he mentions that their influence in spreading yellow fever has been carefully investigated by Dr. Guiteras, Professor of General Pathology and Tropical Medicine in the University of Havana. The yellow fever mosquito, which has been described under several names, must, according to Mr. F. V. Theobald, an authority in matters of classification, be separated from the genus *culex* and placed in a new genus, to which he has given the name of *Stegomyia*, while the species concerned is to be distinguished as *Stegomyia fasciata*. Dr. Guiteras opened an inoculation station in Havana in February 1901, having secured a large number of larvæ of *Stegomyia fasciata*, which were so placed in breeding-jars as to secure a constant supply of complete insects. When a case of yellow fever was reported, a glass jar, containing about 20 female mosquitoes, was taken to the bedside. The patient, passing his hand through a gauze sleeve attached to the mouth of the vessel, and held close around the arm, allowed his hand to rest for a few minutes within the jar, by which time, as a rule, the majority of the insects had filled themselves. Care being taken to prevent their escape, the jar was taken back to the laboratory, where the insects were carefully preserved and fed, and where, in from 12 to 17 days they became pathogenetic. With these infected mosquitoes, Dr. Guiteras succeeded in inoculating eight non-immune persons with yellow fever, and three of the cases so produced terminated fatally. The results, coupled with those of other equally conclusive experiments, show that the prophylaxis of the disease resolves itself into the destruction of the mosquitoes and of their breeding-places. When a case of yellow fever is reported, the chief of the Disinfection Department goes to the house, and requires those interested to designate the apartments in which they desire to be quarantined, which are then fitted with wire-screen doors and windows, so as to make them absolutely mosquito proof. In this way it is hoped to keep already infected mosquitoes within the quarantined area, and to prevent others from getting in. At the same time that the screens are put up the disinfecting squad proceeds to kill the mosquitoes in the building. Every room is carefully gone over, sealed and pasted, and pyrethrum powder is burnt at the rate of a pound to 1,000 cubic feet of air-space. At the same time the three or four contiguous houses are gone over in the same way with pyrethrum powder, with the idea of catching any infected mosquitoes which may have escaped from the infected building. It seems to be established that only a few mosquitoes are usually infected by a single patient, and that these do not wander far from the point of infection. In addition to the measures thus described, precautions are also taken to diminish the number of mosquitoes in the city. The Stegomyia breeds principally in barrels used to collect rain-water for washing purposes, and it is now ordered that all such barrels shall either have a mosquito-proof cover or that the water shall be covered with crude coal oil, and that all yards are to be drained and kept as dry as possible. These methods were commenced on February 16th, 1901, and the effect of them has been that yellow fever was stamped out in less than ten months. The last case in Havana occurred on the 28th of the following September, since when there has not been a single death or case in the city, which has at last been freed from an infection which has been endemic there for 140 years. For the past century there has never before been a day during October or November on which many cases could not have been found in the city, and very few years could be picked out in all this time in which the record for each day during these months did not show several deaths.

